# Inflammaging and Oxidative Stress in Human Diseases: From Molecular Mechanisms to Novel Treatments

**DOI:** 10.3390/ijms20184472

**Published:** 2019-09-10

**Authors:** Li Zuo, Evan R. Prather, Mykola Stetskiv, Davis E. Garrison, James R. Meade, Timotheus I. Peace, Tingyang Zhou

**Affiliations:** 1 College of Arts and Sciences, University of Maine Presque Isle Campus, Presque Isle, ME 04769, USA; 2 Radiologic Sciences and Respiratory Therapy Division, School of Health and Rehabilitation Sciences, The Ohio State University College of Medicine, Columbus, OH 43210, USA; 3 Interdisciplinary Biophysics Graduate Program, The Ohio State University, Columbus, OH 43210, USA

**Keywords:** reactive oxygen species, cardiovascular disease, COPD, cancer, neurodegenerative disease, diabetes, rheumatoid arthritis

## Abstract

It has been proposed that a chronic state of inflammation correlated with aging known as inflammaging, is implicated in multiple disease states commonly observed in the elderly population. Inflammaging is associated with over-abundance of reactive oxygen species in the cell, which can lead to oxidation and damage of cellular components, increased inflammation, and activation of cell death pathways. This review focuses on inflammaging and its contribution to various age-related diseases such as cardiovascular disease, cancer, neurodegenerative diseases, chronic obstructive pulmonary disease, diabetes, and rheumatoid arthritis. Recently published mechanistic details of the roles of reactive oxygen species in inflammaging and various diseases will also be discussed. Advancements in potential treatments to ameliorate inflammaging, oxidative stress, and consequently, reduce the morbidity of multiple disease states will be explored.

## 1. Introduction

Aging is an inevitable and unceasing process that affects everyone indiscriminately. As people age, they become more predisposed to age-related diseases such as heart disease, cancer, respiratory disease, and diabetes, all of which are four of the top seven leading causes of death in the United States of America [1]. Theories of aging fall into two main categories: The programmed theory of aging, which states that people are essentially programmed to age, and the DNA damage theory, in which continuous damage to the DNA over the course of the lifetime leads to aging [2]. While average lifespans of organisms appear to be genetically programmed, DNA damage related with telomere shortening and mutation also play a critical role during aging [3].

The process of aging causes the body to become increasingly susceptible to morbidity and mortality. Aging cells feature a decreased ability to proliferate, and are said to be senescent [4]. Cell senescence stimulates the secretion of pro-inflammatory cytokines that cause chronic inflammation independent from the activation of immune cells [5,6]. This phenomenon of chronic low-grade systemic inflammation that accompanies aging is called “inflammaging”. The same inflammation mechanisms that provide the body protection can contribute to age-related ailments. Inflammation leads to increased levels of reactive oxygen species (ROS) which can induce oxidative stress. It is evident that oxidative stress is a key component in the pathogenesis of chronic inflammation. It has been observed that aged cells have increased levels of oxidant-damaged DNA [7]. Oxidative stress can also lead to the activation of pro-inflammatory pathways in the body [8], which contribute to the pathogenesis of many age-related diseases.

ROS are oxygen-containing and chemically reactive molecules that can be generated within the biological system [9]. The major types of ROS include superoxide (O_2_^•−^), hydrogen peroxide (H_2_O_2_), and hydroxyl radical (^•^OH) [9]. Mitochondria are the most important source of intracellular ROS. O_2_^•−^ are generated when an oxygen (O_2_) molecule gains one electron via electron leakage from the respiratory chain. Thus, disturbance of mitochondria can lead to significant ROS increase [9]. Alternative mechanisms that may contribute to ROS formation include NADPH oxidase (NOX), immune activation, xanthine oxidase (XO), arachidonic acids (AA) metabolism, etc. [9,10]. ROS, when kept at low levels, are essential signaling molecules involved in regulation of cellular or physiological activities such as induction of cell apoptosis, exercise adaptation, and immune response [9,11]. However, redox balance can be disturbed under pathophysiological conditions during inflammation, vigorous exercise, or aging [9,12,13]. The increased ROS accumulation would result in oxidative stress, which cause damage on the primary cellular components including lipids, proteins, and DNA [9]. The accumulation of DNA lesions caused by continuous ROS attack increases the risk of tumorigenesis [14,15]. In addition, ROS over-production may contribute to enhanced inflammatory response, which have been implicated in respiratory diseases such as chronic obstructive pulmonary disease (COPD) and asthma [9]. Antioxidants provide critical protection on the biological system by fighting against oxidative stress. A variety of antioxidants have been identified in the body including superoxide dismutase (SOD), which catalyzes the conversion of O_2_^•^^−^ into H_2_O_2_, as well as glutathione peroxidase (GPX) and catalase, which both catalyze to decompose H_2_O_2_ into H_2_O and O_2_ [9]. In addition to those enzymatic antioxidants, non-enzymatic antioxidant defense plays a critical role in maintaining normal ROS levels, such as vitamin A, C, E, melatonin, and polyphenols [16]. Aging is associated with increased ROS formation and weakened antioxidant defense. Indeed, ROS and inflammaging have mutual stimulatory effects on each other. For example, ROS are shown to activate pryin domain containing-3 protein (NLRP3) inflammasomes, which have been implicated in the development of cardiovascular disease (CVD) and cancer [17]. In order to combat the disparities that plague the growing aging population, it is important to understand the mechanisms of inflammaging in their relation with oxidative stress and their contribution to age-related diseases. The scope of this review is to highlight the underlying mechanisms of inflammaging that contribute to age-related diseases such as CVD, cancer, neurodegenerative diseases, COPD, diabetes, and rheumatoid arthritis (RA) and discuss the potential treatments.

## 2. Inflammaging in Cardiovascular Disease (CVD)

### 2.1. Cardiovascular Diseases in Aging Population

CVD is no longer just a health issue only experienced in western society. CVD is the leading cause of death in the world [18]. As populations age, the risk of contracting CVD increases and is exacerbated by the overall increase in life expectancy during modern times [19,20]. Due to its close correlation with cardiovascular complications, age factor is considered to be critical in cardiovascular status [21]. CVD is characterized by a variety of diseases including myocardial infarction, heart failure, stroke, peripheral arterial disease, arrhythmia, and atrial fibrillation. CVD has been particularly implicated in the development of dementia and loss of daily living function. The syndrome of frailty, which describe increased risk of disability and loss of physiological reserve of older people, is thought to be linked with the development of CVD. In general, CVD is responsible for large levels of mortality, morbidity, loss of function, and disability [22].

### 2.2. General Background and Risk Factors

Decline in vascular function is regarded as one of the key risk factors in the development of CVD (Table 1). Aging causes structural changes in the circulatory system such as the stiffening of arterial walls, which leads to reduction in elasticity and the subsequent development of arteriosclerosis. Several studies including both large and small cohorts have reported that in healthy individuals unaffected by CVD, central arterial stiffness increased with age. Endothelial dysfunction also has been shown to play a crucial role in contributing to the decline in vascular function. It has been consistently shown that vasodilation due to endothelial mediation declines with age. This decline in endothelial function is strongly linked to the pathogenesis of atherosclerosis as well as other manifestations of CVD [23]. Other common dysfunctions include a decrease in perfusion, a lack of vascular growth as well as vascular regression, and vascular remodeling. The heart experiences multiple effects of aging such as diminishment of left ventricular diastolic function, systolic reverse capacity, and a decline in heart rate response to sympathetic modulation. Additionally, contractility and ejection in the left ventricle are exacerbated by age. Hypertrophy can then occur to compensate for the previously listed functional declines leading to more long-term heart issues. Cardiomyocytes are also affected by age through the means of increased apoptosis and increased susceptibility to stress. Importantly, stem-cell induced cardiomyocyte regeneration decreases substantially with age. The heart and vasculature are not independent entities and therefore changes in the heart can affect the vasculature and vice versa [21].

### 2.3. Oxidative Stress and Inflammaging in Cardiovascular Aging

**Figure 1:**
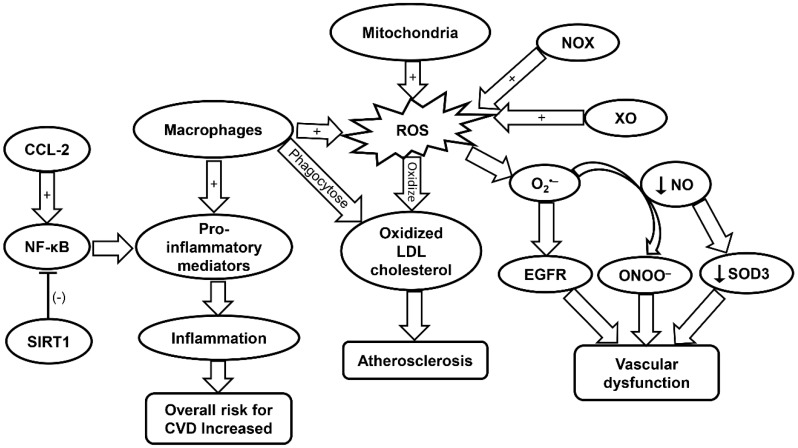
Schematic summarizing the molecular mechanisms contributing to CVD progression. CCL-2, chemokine (C-C motif) ligand 2; CVD, cardiovascular disease; EGFR, epidermal growth factor receptor; LDL, low density lipoprotein; NF-κB, nuclear factor kappa-light-chain-enhancer of activated B; NOX, NADPH oxidase; ROS, reactive oxygen species; SIRT1, sirtuin-1; SOD, superoxide dismutase; XO, xanthine oxidase.

Oxidative stress has serious implication in the development of CVD. As previously mentioned, atherosclerosis is common in the aging population and can be exacerbated by oxidative stress. Low density lipoprotein (LDL) cholesterol can become oxidized in the initial stage of atherogenesis, which leads to the activation of endothelium and initiation of immune system response. Inflammatory cells such as monocytes and T cells adhere and migrate into the arterial intima. The oxidized LDL is then phagocytosed causing macrophages to release ROS and pro-inflammatory markers that subsequently lead to further LDL oxidation (Figure 1) [25]. This cycle leads to the progression of atherosclerosis (Table 1) [26].

Dysfunction of the endothelium can be attributed to ROS produced via the electron transport chain in the mitochondria. Specifically, mitochondrial ROS (mtROS) and NOX-ROS have been demonstrated to regulate each other in a positive feedback loop [26]. Under a physiological state, nitric oxide (NO) is a key regulator of endothelial function. However, under the condition in which there is an increase in ROS levels such as O_2_^•−^, NO be oxidized and become a powerful oxidizer, peroxynitrite ONOO^−^, which leads to additional oxidation and cell damage [26]. NOX enzymes expressed in vascular tissue produce ROS that are linked to CVD (Figure 1) [26]. Specifically, NOX4 is implicated in the regulation of vascular smooth-muscle cells (VSMCs), fibroblasts, and differentiation and migration of cardiac cells [100]. In the presence of transforming growth factor (TGF)-β, NOX4 produces a large amount of ROS. Smooth muscle (alpha) actin can then be activated by this increased NOX4-ROS production through inhibition of MKP-1 phosphatase. This leads to the phosphorylation of serum response factor and the subsequent binding to myocardin-related transcription factor by means of p38 mitogen-activated protein kinase (MAPK), resulting in VSMC differentiation. However, NOX4 can induce negative effects when overexpressed due to increased production of H_2_O_2_ [26].

Xanthine oxidase (XO) is also involved in the regulation and dysregulation of the endothelium. ROS produced by XO has been shown to react with O_2_^•−^ to produce OONO^−^ and cause subsequent cellular damage [26]. Additionally, XO-derived O_2_^•−^ has been implicated in the progression of pulmonary hypertension. It has been demonstrated that the ROS produced by XO directly interacts with epidermal growth factor receptor (EGFR) thereby inducing vascular remodeling and CVD (Figure 1 and Table 1). Lipoxygenase (LO) also plays a role in the development of CVD [26]. Specifically, 5-LO is of interest due to its involvement in activating inflammatory cells. 5-LO can be upregulated in response to oxidative stress while downregulation of 5-LO shows positive effects on myocardial infarction. Yet, the associated mechanisms remain to be elucidated [26,101]. Myeloperoxidase (MPO) has been demonstrated to promote the oxidation of LDL, and is therefore implicated in CVD. Furthermore, MPO can promote endothelial dysfunction through limiting the availability of NO (Table 1) [27].

SOD has also been implicated in vascular disease in response to oxidative stress. SOD is the main enzyme responsible for dismutating O_2_^•−^ into H_2_O_2_. However, it remains controversial as to whether SOD activity is increased or decreased with aging [102]. Particularly, SOD1 plays a key role in maintaining the endothelial function by protecting the NO availability. In atherosclerosis, SOD1 has been shown to decrease the levels of O_2_^•−^, to maintain normal vascular function. This is evident in the aging populations who are constantly exposed to oxidative elements such as angiotensin II and lipopolysaccharide [28]. Lack of SOD ultimately leads to heightened levels of vasoconstriction and endothelium malfunction. Cells deficient in SOD2 exhibit an increase in mitochondrial damage in mice. Moreover, aged mice heterozygous for SOD2 were found to exhibit impaired vasorelaxation with high levels of ROS and radical formation in mitochondria [28]. SOD3 is important in the regulation of radical NO following its release from endothelial cells. A decrease in NO production can lower expression of SOD3 resulting in more cellular damage (Figure 1). SOD3 has also been shown to decrease age-related vascular dysfunction in a rat model (Table 1) [28].

In cases of coronary artery disease and atherosclerosis, GPX-1 activity is considered as a marker for both disease and levels of ROS [103]. GPX1 protects erythrocytes from oxidation and subsequent damage [104]. In one study conducted by Wickremasinghe et al., it was demonstrated that there was an inverse relationship between the activity of erythrocyte GPX-1 and coronary artery disease, similar to these in atherosclerosis [103]. It was reported in another study that there was a positive relationship found between GPX3 activity and CVD in aging populations affected by atrial fibrillation [103]. A deficiency in GPX3 ultimately leads to an increase in an oxidative and prothrombotic environment due to impaired ROS inactivation [105]. Platelet activation was the major marker for GPX3 activity monitored by thromboxane B2 excretion. Additionally, in aging populations, the overall activity of GPX3 declines with aging. However, further study needs to explore the exact mechanism associated with such finding [103].

Cytokines are key players in the progression of atherosclerosis and CVD. For example, interferon (IFN)-γ increases the expression of the pro-inflammatory phenotype (M1) of macrophages. This leads to increased arterial plaque formation and additional cell apoptosis in cells leading to lipid expulsion into adjacent plaque areas. In addition to IFN-γ, interleukin (IL)-1β can also induce the M1 macrophage phenotypes in an auto-inflammatory fashion or act as an activator to other pro-inflammatory genes. Furthermore, oxidized LDL induces increased expression of IL-1β, leading to higher levels of inflammation within the atherosclerotic plaque region. Chemokine (C-C motif) ligand 2 (CCL-2) cytokine is involved in the development of atherosclerotic plaques through the protein kinase C/extracellular regulated protein kinases (ERK)1/nuclear factor kappa-light-chain-enhancer of activated B (NF-κB) signaling pathway (Table 1). It has been demonstrated that cells lacking CCL-2 produce smaller plaques (Figure 1) [106]. C-reactive protein (CRP), which is an inflammatory mediator commonly found in CVD, has been demonstrated to increase oxidation stress in endothelial cells. Yet, there is conflicting evidence that CRP is merely a marker for oxidative stress rather than a causative agent [30].

### 2.4. Roles of Inflammaging in CVD

Inflammaging in CVD is caused by chronic inflammation, which can be traced to a variety of sources. In aged individuals, the tissues surrounding the cardiovascular system are chronically inflamed leading to an increased production of reactive species. Such age-associated chronic vascular age is thought to be mediated by inflammatory secretome produced from aging VSMC and endothelial cells [24]. Additionally, cell and immune senescence can contribute to inflammaging. It was suggested that senescent leukocytes maybe related with the formation of atherosclerotic plaques [24]. Large amounts of microRNAs have also been implicated in inflammaging [107]. This is important because microRNA modulates mitochondrial activity, such as proliferation of dysfunctional mitochondria, thus, contributing to inflammaging and furthering susceptibility to CVD [108]. Lastly, epigenetic alterations in gene transcription are associated with oxidative stress in the aging cardiovascular system. Through chromatin remodeling, atypical cellular expression of inflammatory genes leads to aberrant molecular pathways therefore increasing vulnerability to CVD [109].

### 2.5. Senescence Cells and CVD

Cell senescence is a phenomenon in which cells cease to divide in response to stressors [110]. Age-related senescence can lead to increased expression of proinflammatory genes that is linked with chronic inflammation [24]. Telomerase is essential for proper endothelial and vascular smooth muscle cell function since deletion of the gene has been shown to increase cell senescence [24,111]. In atherosclerosis plaques, there is increased telomeric exhaustion due to the reduced activity of telomerase in plaques [24]. In CVD, aging causes progressive accumulation of dysfunctional cells induced by telomere-dependent senescence [24]. In fact, in the presence of senescent endothelial cells, VSMCs start to proliferate leading to pathogenic vascular remodeling and can also calcify in the presence of cell senescence [24]. The gene SIRT1 is responsible for controlling calcification of VSMCs and reducing pathogenic effects of senescent VSMCs [24]. Sirtuin-1 (SIRT1) protein coupled with Rel/p65 can act as a negative regulator of NF-κB transcriptional activity [31]. The NF-κB transcriptional factor is important for the regulation of gene pathways responsible for inflammation as well as cell adhesion and proliferation. As such age-related NF-κB activation was linked with systemic inflammation and impaired vessel dilation function (Figure 1 & Table 1) [31].

### 2.6. Immunosenescence and CVD

Immunosenescence is characterized by a decline in immune potential due to age. Thus, it is considered an additional risk factor for CVD as well as a known contributor to inflammaging. Senescent macrophages have been shown to be a direct cause of atherosclerotic plaques [24]. Oxidative stress can also increase the load on the innate immune system which increases the incidence of chronic inflammation and induce atherosclerosis [112]. Leukocyte telomere length (LTL) when shortened is associated with increased senescent epithelial cells and fibroblasts, which contributes to the progression of CVD (Table 1) [32]. Senescent leukocytes themselves can be a marker for CVD as they can display the senescence-associated secretory phenotype (SASP), another cause of CVD [24]. In the elderly, monocytes have been shown to have decreased activity regarding NO production, which is likely correlated with vascular dysfunction [113].

Immunosenescence also plays a role in regulatory impairment of fibroblasts and consequently, remodeled cardiac morphology through upregulation of cytokines [114]. Reduced lymphocyte counts were found to be characteristic of advanced heart failure. Marked reduction of B-cells and T cytotoxic cells, as well as the increase in T helper cell differentiation and aging were observed in patients with heart failure [115]. Senescent immune cells are commonly located in atherosclerotic plaques. Specifically, CD4^+^ and CD28^null^ cells have been found in coronary plaques; while CD8^+^ and CD28^null^ are in the bloodstream in patients with coronary complications. The aforementioned senescent T cells contribute to the pathogenesis of various CVD through the release of effector molecules, leading to upregulation of cytokines including IFN-γ (Table 1). Furthermore, hypertensive patients show increased levels of cytokines such as IFN-γ and tumor necrosis factor (TNF)-α in the peripheral blood. Further research is required, however, to elucidate a firm link between T-cell senescence and CVD prevalence [29]. These data taken together suggest the increased risk of CVD in the elderly as a consequence of immunosenescence. Furthermore, in contrast with control subjects, chronic heart failure patients showed a heightened level of immunosenescence. Increases in T-lymphocyte differentiation along with high levels of IL-6 accelerates disease progression via attenuated immune reaction and accelerated aging of patients’ immune system [116].

## 3. Inflammaging in Cancer

### 3.1. Cancer in Aging Population

Torre et al. provided statistics that shows 14.1 million new cancer cases and 8.2 million cancer-related deaths in 2012. The leading cause of cancer-related death in men is lung cancer from both less-developed and more developed countries [117]. Breast cancer remains the most common cancer in women, with more than 1 million new cases each year [118]. Torre et al. attributes the increasing occurrence of cancer to increased cancer risk factors as well as the growth of the aging population [117]. According to Li et al., 60% of all cancer diagnoses and 70% of cancer-related mortalities are found in the population aged 65 or older, and it is estimated that by the year 2030, 70% of all cancer diagnoses will occur in this aging group [119].

### 3.2. Oxidative Stress in Cancer

**Figure 2:**
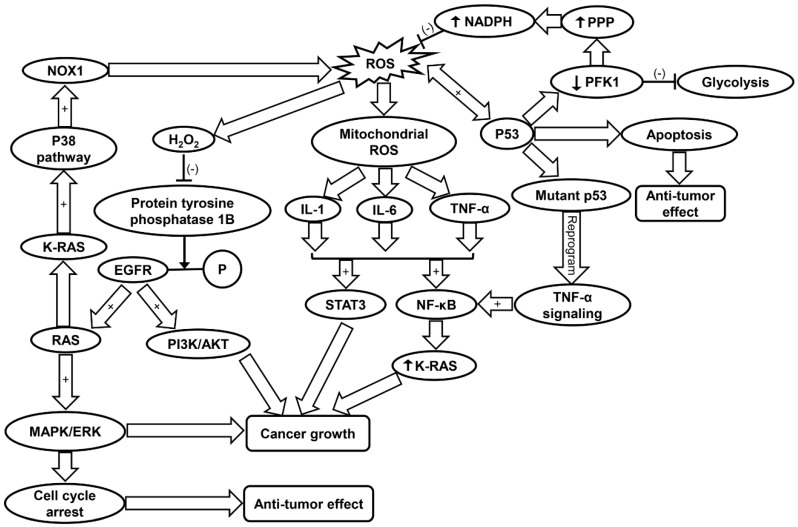
Schematic summarizing the molecular mechanisms contributing to cancer progression. EGFR, epidermal growth factor receptor; IL-1, interleukin-1; IL-6, interleukin-6; MAPK/ERK, mitogen-activated protein kinase/extracellular regulated protein kinases; NF-κB, nuclear factor kappa-light-chain-enhancer of activated B; NOX1, NADPH oxidase 1; PFK, phosphofructokinase; PI3K/AKT, phosphatidyl inositol 3-kinase/protein kinase B; PPP, pentose phosphate pathway; ROS, reactive oxygen species; SIRT1, sirtuin-1; STAT3, signal transducers and activators of transcription 3; TNF-α, tumor necrosis factor-α.

Cancer cells exhibit an altered metabolism to compensate for higher energy demands due to increased growth and proliferation [120]. As a result, cancer cells produce more ROS than typical cells to maintain normal subcellular activities such as signal transduction and gene expression [120]. Increased mitochondrial ROS levels promote tumorigenesis through mtDNA mutagenesis and redox signaling [14]. Studies have corroborated that ROS drive tumorigenesis through oncogenic cell signaling pathways (Table 1) [40]. Conversely, excessive ROS formation can also trigger cell death in cancer cell lines [121,122]. Frequently involved in cancer are MAPK pathways [123]. Two pathways commonly associated with cancer are the phosphatidyl inositol 3-kinase/protein kinase B (PI3K/AKT) and RAS-MEK-ERK pathways, which promote cell survival, proliferation, inflammation, metabolism, and nutrient uptake [40,124,125]. H_2_O_2_ inactivates protein tyrosine phosphatase 1B, which inhibits dephosphorylation of EGFR thus promoting downstream PI3K/AKT and RAS-MEK-ERK (ERK/MAPK) pathways [40]. Ras, a GTPase is frequently involved in tumorigenesis by activation of MAPK pathways and transcriptional regulation. K-Ras specifically is involved in activation of c-Jun N-terminal kinase (JNK), ERK, and p38 signaling pathways, the latter of which also contributes to ROS generation via NOX1 [126]. The PI3K/AKT pathway is prevalent in cancer development. Research in recent years has looked into inhibition of this pathway in various cancer populations [127,128]. Additionally, it has been shown that high-intensity constitutive activation of the MAPK/ERK pathway triggers cell cycle arrest and prevents cell transformation in cells expressing RAS [129,130]. However, this conflicts with the previous findings that the activation of ERK/MAPK can promote tumor cell growth [131]. It was concluded that ERK/MAPK’s tumor suppressor/oncogene activity depends on the strength of its activity and senescence-associated protein degradation [129]. ROS-dependent p38 MAPK activation has been shown to lead to the loss of self-renewal, differentiation, and tumor-initiating capacity in glioma-initiating cells [41]. Conversely, blockage of p38MAPK signaling was found to reduce cancer growth in head and neck squamous cell carcinoma [42]. Thus, it can be suggested that ROS may either enhance, inhibit or regulate tumorigenesis via activation of MAPK pathways to different degrees in various cancers (Figure 2 and Table 1).

Although ROS can contribute to cancer progression, oxidative stress-related mitochondrial dysfunction can trigger cell apoptosis [132]. Cancer cells protect themselves from increased levels of ROS in part by upregulating antioxidant transcription factors such as nuclear factor erythroid 2-related factor 2 (NRF2) via oncogene expression (K-Ras, B-Raf, c-Myc) [132]. Furthermore, ROS have a direct role in the expression of NRF2. Oxidation of cysteine residues on Kelch-like ECH-associated protein 1 (KEAP1) prevents degradation of NRF2 and allows it to act as a transcription factor for antioxidant genes [40,133]. NRF2 is instrumental in regulating expression of GSH, GPX, as well as multiple glutathione S-transferases [133,134]. NRF2 also promotes increased activation of the pentose phosphate pathway (PPP), which leads to production of NADPH [43]. NADPH is involved in the regeneration of reduced GSH from GSSG, thereby maintaining cellular antioxidant levels [40]. Finally, NRF2 can accelerate cancer cell proliferation via direction of anabolic purine synthesis pathways (Table 1) [43].

It has been shown that p53, a tumor suppressor, also has a significant role in antioxidant response. p53 induces expression of apoptosis factor TP53-induced glycolysis and apoptosis regulator (TIGAR) and down-regulates phosphofructokinase (PFK)-1 by lowering fructose-2,6-bisphosphatase, inhibiting glycolysis and lowering intracellular ROS levels [135]. The blocking of glycolysis and simultaneous shuttling metabolites to the PPP leads to production of NADPH [135]. p53 promotes either pro-oxidant activity under oxidative stress or antioxidant activity under normal conditions [45]. The pro-oxidant activity of p53 under high stress leads to apoptosis (Table 1) [46]. One study suggests that p53 and ROS have a reciprocal relationship in the coordination of apoptosis in colorectal cancer cells [136]. Another study found that ROS-dependent activation of JNK promotes apoptosis activity of p53 (Figure 2) [44]. Additionally, reduction of DNA damage, ROS levels, and p53 expression by antioxidant treatment increases tumor cell proliferation in cancer cells [137].

ROS can induce mutagenesis that leads to cancer via mutagenic DNA lesions [15]. Among DNA biomarkers of oxidative stress are 8-oxo-7,8-dihydro-2’-deoxyguanine (8-oxodG) and 8-nitroguanine, both of which have been observed in inflammatory-cancer tissues in various infectious and non-infectious agents [15,138]. 8-oxodG mutations are prevalent in the mutated p53 gene, further linking oxidative stress with cancer [139]. Mitochondrial DNA (mtDNA) mutations can lead to increased ROS generation through disruptions of electron transport chain [140]. However, there have been conflicting reports of whether or not mtDNA mutations can facilitate cancer development [14,141].

### 3.3. Inflammaging in Cancer

A major hallmark of cancer is the presence of inflammation. Inflammation contributes to tumorigenesis via enhancement of proliferative and survival signaling, as well as the facilitation of angiogenesis, invasion, and metastasis [48,49]. Inflammaging features increased plasma levels as well as enhanced cellular production of pro-inflammatory cytokines such as IL-1, IL-6, and TNF-α [47]. This chronic, low-level inflammation becomes more prominent with age, and contributes to age-related diseases [50]. Chronic low-level inflammation exposes cells to constant increased levels of ROS, which endangers cellular genomes to mutation (Table 1) [46].

One of the most prevalent immune cells involved in immune system’s reaction to cancer is the neutrophil. Neutrophils have both pro and anti-tumor development roles, and the number of neutrophils in circulation increases with tumor development, eventually making up as much as 90% of the leukocyte count [57]. It has also been established that tumor-associated neutrophils in murine models can switch their phenotype from a tumor supportive to a tumor cytotoxic phenotype. Closely related to neutrophils are granylocytic-myeloid derived suppressor cells (G-MDSCs), which contribute to tumor growth and progression by producing ROS to suppress CD8^+^ T-cells (Table 1) [57]. Circulating neutrophils in cancer have been split into three distinct sub-populations: high density neutrophils, which are more prevalent in early-stage tumors and have an overall anti-tumor effect, low density neutrophils, which become dominant in late-stage tumors and have an overall pro-tumor effect, and G-MDSCs [57]. Similarly, monocytes in murine models have also shown differences in how they respond to cancer. In breast cancer, inflammatory monocytes were observed to promote cancer, while patrolling monocytes appeared to have an anti-tumor role. While the total number of monocytes increases significantly in late-stage cancer, only the inflammatory blood monocytes experience transcriptional alterations. The changes in inflammatory blood monocytes are associated with the down regulation of T-cell migration, antigen-cross-presentation, interferon response, and cytokine stimulus [142].

ROS regulate inflammation through activation of pro-inflammatory cytokines and NLRP3 inflammasome [56,143]. Specifically, mitochondria-derived ROS formation is found to induce the upregulation of IL-1, IL-6, and TNF-α [56]. These cytokines have been shown to activate NF-κB and signal transducers and activators of transcription 3 (STAT3), both of which have been implicated in cancer growth [51,52,53]. TNF-α has been found to induce tumorigenesis via generation of ROS and subsequent damage of DNA [49]. Mutant p53 has been shown to reprogram TNF-α signaling to favor NF-κB activation over the ASK1/JNK pathway [54]. NF-κB can increase oncogenic K-Ras levels in a positive feedback loop, further correlating chronic inflammation with cancer progression (Figure 2 and Table 1) [55]. Furthermore, most target genes of IL-6 are involved in cell survival and proliferation, thereby contributing to cancer cell growth [144]. Inhibition of IL-1 suppressed growth of head and neck squamous carcinoma cells, indicating its role in tumorigenesis [145].

Evidence has shown that ROS are important in the activation of the NOD-like receptor family, NLRP3 inflammasome [17]. The NLRP3 inflammasome has been identified as a putative oncogene in non-small cell lung cancer genomic analyses, however, recent evidence suggests that it also has tumor suppressor function [146]. It has been shown that the NLRP3 inflammasome inhibits tumor growth in colorectal cancer [147]. Downregulation of the NLRP3 inflammasome was also found in human hepatocellular carcinoma tissue [148]. Conversely, the NLRP3 inflammasome was able to inhibit chemotherapeutic agents and promote tumorigenesis [149]. In epidermoid carcinoma-derived cells, inhibition of NLRP3 inflammasome led to cell death. This result indicates that the activation of NLRP3 inflammasome contributes to the survival of head and neck squamous cell carcinoma [150].

## 4. Inflammaging in Neurodegenerative Diseases

### 4.1. Inflammaging in Neurodegeneration

**Figure 3:**
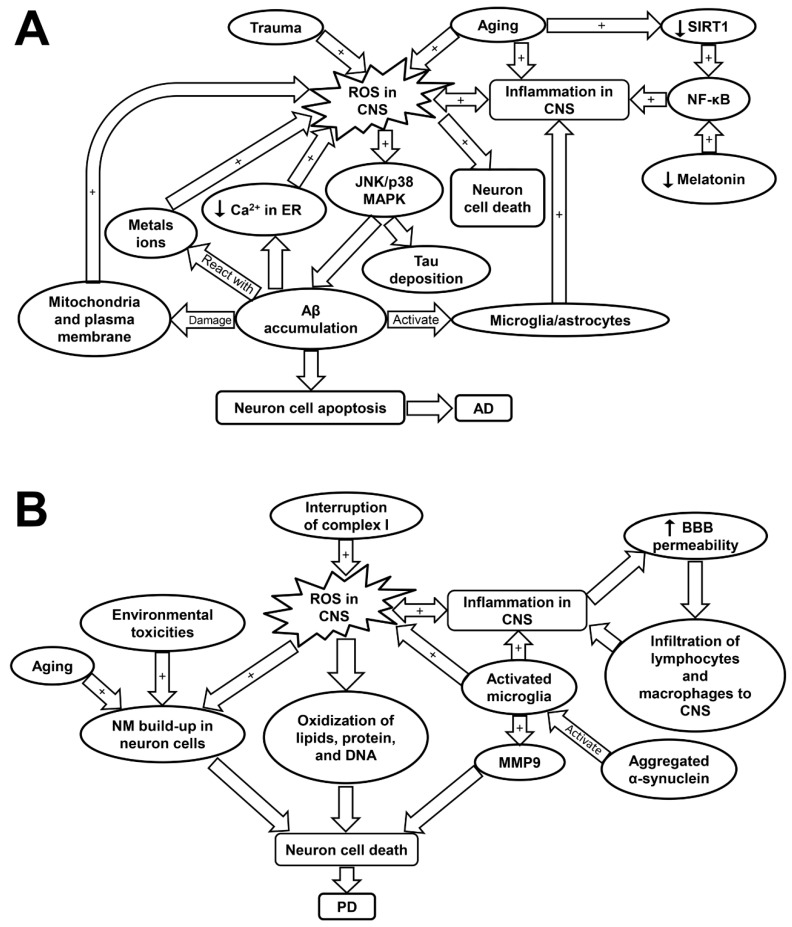
Schematic showing the molecular mechanisms underlying Alzheimer’s disease (**A**) and Parkinson’s disease (**B**). AD, Alzheimer’s disease; Aβ, amyloid beta plaques; BBB, blood brain barrier; CNS, central nervous system; JNK/p38 MAPK, c-Jun N-terminal kinase/p38 mitogen-activated protein kinase; MMP9, matrix metalloprotease 9; NF-κB, nuclear factor kappa-light-chain-enhancer of activated B; PD, Parkinson’s disease; ROS, reactive oxygen species; SIRT1, sirtuin-1.

Advanced age is considered a major risk factor of cognitive dysfunction and neurodegenerative diseases such as Alzheimer’s disease (AD) and Parkinson’s disease (PD) [60,62]. As people age, there is significant increase of oxidative stress within the nervous system, leading to reduced regenerative capacity and functional decline of the nerves [60,61]. Specifically, voltage-gated potassium (K^+^) channel sub-family B member 1 (KCNB1) is shown to be subjected to moderate oxidation in aged people, causing hippocampal functional impairment. In AD or following a trauma, the oxidation of KCNB1 is aggravated, resulting in marked neurodegeneration (Figure 3A and Table 1) [60]. Inflammaging is not only restricted to the blood and bone marrow, but also detected in the human central nervous system [151]. Sustained inflammation was found in the intact aging nerves with increased levels of CC chemokine ligand 11 (CCL11) and monocyte chemoattractant protein 1, as well as amplified macrophage infiltration. Elevated CCL11 interrupts the differentiation of Schwann cells, which contribute to reduced regenerative capability of the aged nerves (Table 1) [61]. Increased levels of inflammatory cytokines including TNF-α, IFN-γ-induced protein 10 (IP-10), and IL-8 have been observed in the cerebrospinal fluid (CSF) of aging individuals. The immune cell profile is characterized by a shift from T helper cell 1 (Th1) to non-Th1 inflammatory phenotype [151]. This age-dependent inflammatory phenotype shift was found to be heightened in AD and multiple sclerosis (MS) [151]. While MS is mostly diagnosed at an early-to-middle age, the progenitor cell senescence related with premature cell aging has been indicated to play a key role in its pathogenesis [152].

### 4.2. Inflammaging and Oxidative Stress in Alzheimer’s Disease (AD)

AD is the leading cause of dementia among the aging population with high morbidity and mortality [63]. The disease is primarily characterized by memory loss and cognitive impairment due to progressive neurodegeneration [153]. Individuals with advanced age, obesity, diabetes, or hypercholesterolemia are more likely to be affected by AD [154,155]. The pathogenesis of AD is linked with intracellular deposition of Tau neurofibrillary tangles (NFT) and extracellular accumulation of amyloid beta (Aβ) plaques (Table 1) [62]. Aβ plaques can mediate cellular apoptosis through the activation of Bcl-2-associated death promoter and caspase cascades (Figure 3A) [62]. It is also hypothesized that Aβ plaques contributes to Tau phosphorylation. The phosphorylated Tau loses its affinity to microtubules and aggregates to form the detrimental NFT [156]. However, other studies suggested that the phosphorylation of Tau occurs following its aggregation [156]. Aβ peptide, the major component of Aβ plaque, is the proteolytic product of amyloid precursor protein (APP); yet the physiological function of APP remains controversial [156,157]. Studies have indicated that mutation in APP protein is associated with the abnormal deposition of Aβ [158]. Interestingly, Aβ is not only found in AD patients, but also in the CSF of healthy individuals, which, however, can be efficiently cleared in the healthy body especially during sleep [159]. Therefore, lack of sleep could have a major role in AD development, which is potentially correlated with melatonin dysregulation. Melatonin has been shown to assist in Aβ clearance as well as inflammation suppression [160,161].

ROS-induced oxidative stress plays a critical role in the pathogenesis of AD (Table 1) [16]. Although it remains debated whether increased ROS formation is the primary cause or a consequence of AD, severe oxidative stress was detected in the early stage of AD even prior to the marked Aβ accumulation [162]. It has been shown that increased ROS production contributes to Aβ accumulation and Tau hyper-phosphorylation via the activation of JNK/p38 MAPK pathways (Table 1) [16]. The deposition of Aβ disturbs Ca^2+^ balance in endoplasmic reticulum (ER) as well as damages mitochondrial and plasma membrane, which exacerbates ROS production [62]. The Aβ plaque-activated microglia and astrocytes also perform as primary sources for ROS in AD [12]. In addition, Aβ plaques contain high levels of metal ions such as copper and iron, which facilitate the formation of H_2_O_2_ through the reaction between Aβ plaques and the metal ions [63]. The excess ROS accumulation has been implicated in the death of neuron cells via its attacking to DNA, lipids, or protein leading to neuron apoptosis (Figure 3A and Table 1) [63].

Interestingly, as the disease progresses, ROS-induced oxidative damage decreases with Aβ deposition [63]. ROS formation was induced by over-activation of inflammatory responses in the initial stage of the disease. Therefore, inflammaging may be an initiator and an exacerbating factor in AD development (Figure 3A) [63]. The reduction in oxidative damage is potentially associated with the decline in adaptive immune activity during the disease progression [63]. An examination of the immune profiles in AD patients revealed a marked decrease in CD19^+^ and CD3^+^ lymphocytes, a decrease in CD9^+^ cells, and a slight elevation in CD4^+^ cells. The ratio of CD4^+^/CD8^+^ cells was not significantly altered. These findings suggest an attenuated adaptive immune response in AD patients characterized by a decline in B- and T- cell numbers as compared to those in healthy individuals [163]. On the contrary, the innate immune response was over-activated by Aβ deposits in astrocytes and microglia in order to clear up the aggregate peptides. However, this would also result in the over-production of pro-inflammatory cytokines and ROS that may be responsible for neurodegeneration in AD [63]. Fibrillar Aβ has been shown to trigger the release of pro-inflammatory cytokines via the activation of toll-like receptors (TLRs). Certain inflammatory proteins including clusterin, apolipoprotein E, and activated complement proteins were detected within the Aβ lesions [164]. Moreover, it was found that the offspring of late-onset AD patients displayed a stronger innate immunity responsiveness than normal people, further linking inflammation to the pathology of AD [164].

NF-κB is a redox-sensitive transcription factor that are implicated in controlling of gene expression related with pro-inflammation, antioxidants, and pro-apoptosis. Evidence has shown that NF-κB is highly activated in the AD brain, leading to pro-inflammatory gene expression [12,165]. The activation of NF-κB pathways is potentially induced by oxidative stress, over-activated microglia, and reduced SIRT1 expression (Figure 3A) [166]. SIRT1 plays a critical role in maintaining the normal neuron function, but its expression was found to decrease with age [166]. The low SIRT1 expression in neuron cells is associated with cognitive impairment and memory loss. SIRT1 was reported to inhibit Aβ formation by triggering peroxisome proliferator-activated receptor-γ coactivator 1alpha (PGC-1α). SIRT1 can also inhibit NF-κB pathways, and thus attenuate inflammatory response associated with AD [166]. Melatonin, apart from its circadian roles, is another crucial regulator that can suppress NF-κB activity (Figure 3A) [160]. Indeed, melatonin and SIRT1 was reported to have mutual effects on each other and have overlapping roles in anti-inflammatory effects such as suppressing the activation of NF-κB cascades and blocking TLR-4 signaling. Therefore, it is likely that age-associated melatonin and SIRT1 deficiency is significantly involved in both inflammaging and AD development (Table 1) [160].

### 4.3. Inflammaging and Oxidative Stress in Parkinson’s Disease (PD)

PD is the second most prevalent neurodegenerative disorder after AD. About 0.014% of the total population in high-income countries are affected by PD, while this rate was raised to 1.6% over the age of 65, and to 3% over 80 [62,67,167]. This incurable disease is manifested by abnormal motor behaviors including uncontrollable tremor, postural instability, bradykinesia, muscle rigidity, and depression [69]. The primary causes of death among PD patients are pneumonia, cerebrovascular disease, and CVD [67]. Only ~10% of PD cases show family history; while the remaining 90% of PD have no clearly defined genetic cause [168]. The pathogenesis of PD is linked with progressive loss of dopaminergic neurons in the substantia nigra pars compacta (SNpc) area of the brain and the intracellular deposition of misfolded α-synuclein (Table 1) [66,67].

ROS formation has been considered to be involved in PD progression [62]. A recent study by Carballo-Carbajal et al. proposed that the neurons which contain high levels of pigment neuromelanin (NM) are especially susceptible to PD-associated neurodegeneration [169]. The physiological roles of NM remain controversial, but may be associated with sequestering of toxic metals, pesticide, and ROS. Thus, the continuous buildup of NM may be linked with aging, exposure to environmental toxicities, and oxidative stress [169]. Specifically, ROS-induced dopamine auto-oxidation appears to cause NM formation [155]. Neurodegeneration in PD is triggered when the NM levels reach a threshold that can interfere the normal neural function [169]. NM-laden cells displayed high levels of oxidative stress and disturbed mitochondrial respiration, which may be responsible for the substantial cell death observed in this study [169]. Furthermore, complex I inhibition and mitochondria-induced ROS are suggested to account for the majority of neuron cell loss in PD as dopaminergic neurons are particularly susceptible to oxidative stress (Table 1) [68,170,171]. It was found that mitochondrial function and complex I activity was interrupted in the striatum and cortex of the rats with PD induced by rotenone treatment (Figure 3B) [68]. Elevation in ROS levels can lead to oxidation of lipid, protein, and DNA, as well as induce neuron cell apoptosis [62]. Several common mutations related with PD such as leucine-rich repeat kinase 2 (LRRK2), PTEN-induced putative kinase 1 (PINK1), and parkin have been implicated in the disturbance of redox balance and mitochondrial dysfunction in neuron cells [62]. Specifically, the activation of LRRK2 is associated with increased oxidative stress and amplified inflammatory response, which may underlie the mechanisms of the disease progression [168]. 

In PD, activated microglial cells play a critical role in neuroinflammation and perform as an important source of ROS, nitrogen species, matrix metalloprotease (MMP), and pro-inflammatory cytokines [70]. The expression of MMP, especially MMP-9 are upregulated in response to brain injury and they have a crucial role in mediating neuron cell migration and apoptosis [172]. The activation of microglia may be induced by pro-inflammatory cytokines including IFN-γ and TNF-α or by the triggering of TLRs (Figure 3B and Table 1) [69,70]. Importantly, microglial cells can be activated with either inflammatory (M-1 like) phenotype, which is neurotoxic, or anti-inflammatory (M-2 like) phenotype, which is neuro-supportive [70,173]. Neurodegeneration occurs when M-1 phenotype is the predominant phenotype acquired by microglia, as in the case of PD [70]. Aged microglia are inclined to develop M1-like phenotype because of the sustained cerebral exposure to low-grade inflammation [71]. T cells also play an essential role in the neuron loss of PD. This is based on the fact that mice with T-cell deficiency demonstrated a significantly attenuated dopaminergic neurodegeneration [174]. Furthermore, studies have shown that the blood brain barrier (BBB) permeabilization is favored by microglia- and inflammaging-induced pro-inflammatory cytokines. This allows for infiltration of T cells and leukocytes into the CNS, as evidenced by the post-mortem examination of PD patients [69,70,71]. Thus, the alterations of peripheral blood lymphocytes could have a marked impact on neurodegeneration and microglial activation (Figure 3B and Table 1) [70]. Evidence has shown that PD patients exhibited a decline in circulating CD4^+^ T cells. Specifically, the levels of Th2, Th17, and regulatory T cells are decreased; while T naive cells show a preferential differentiation into Th1 lineage, which is accompanied by raised levels of IFN-γ and TNF-α [70]. Moreover, it was shown that inflammaging is associated with brain cell death via inflammasome activation, which could be a precursor contributing to the development of PD [175].

## 5. Inflammaging in Chronic Obstructive Pulmonary Disease (COPD)

### 5.1. Introduction of COPD

**Figure 4:**
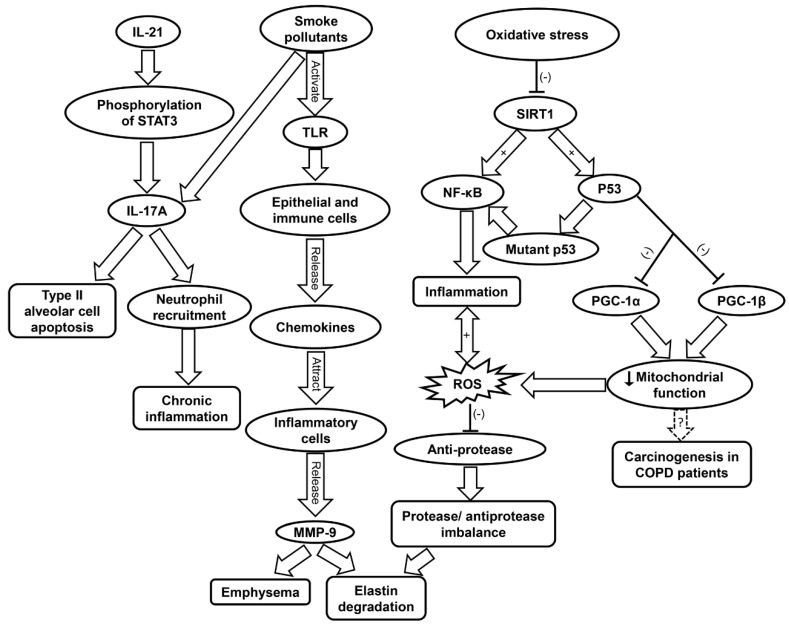
Schematic showing the molecular mechanisms contributing to COPD progression. COPD, chronic obstructive pulmonary disease; IL-17A, interleukin-17A; IL-21, interleukin-21; MMP9, matrix metalloprotease 9; NF-κB, nuclear factor kappa-light-chain-enhancer of activated B; PGC, peroxisome proliferator-activated receptor-γ coactivator; ROS, reactive oxygen species; SIRT1, sirtuin-1; STAT3, signal transducers and activators of transcription 3; TLR, toll-like receptor.

As lungs age, natural antioxidant response and intracellular signaling deteriorate, which contributes to inflammaging [176,177,178]. Aging lungs display features similar to symptoms observed in COPD, such as oxidative stress and chronic inflammation [179]. COPD is most prevalent in middle-aged and elderly populations, and is associated with increased oxidative stress and inflammation [180,181]. Cigarette smoke and toxic gases contribute to COPD by induction of abnormal lung inflammation (Table 1) [74]. This occurs when pattern recognition receptors (PRRs) are triggered by cigarette pollutants. Following the activation of PRRs such as TLRs, epithelial cells and immune cells release chemokines that attract the inflammatory cells to the site of inflammation which include Th1 cells, type 1 cytotoxic T cells, monocytes, and neutrophils [182,183]. These inflammatory cells release proteases such as MMP-9, which contribute to emphysema and elastin degradation. Additionally, macrophages release TGF-β which induces tissue remodeling. Increased oxidative stress in the small airways causes protease/anti-protease imbalance via the inactivation of anti-proteases and upregulation of pro-inflammatory mediators, which underlies the pathogenesis of COPD (Figure 4 and Table 1) [75].

### 5.2. Inflammatory Cells Involved in COPD

Neutrophils, macrophages, and CD8 T lymphocytes are thought to be the predominant inflammatory cell types involved in COPD [183]. Specifically, neutrophils contribute to tissue damage and inflammation response in COPD via degranulation and ROS generation [76]. A recent study found that depletion of neutrophils via anti-Ly6G antibodies inhibited emphysema development as well as small airway remodeling [184]. Additionally, significantly increased M1 and M2 polarization have been seen in COPD models [185]. The M1 phenotype is the classical pro-inflammatory profile of macrophages; while M2 phenotype is associated with tissue remodeling and repair, and is considered the alternative phenotype [185]. Inflammation triggers M1 polarization but not M2 polarization, further contributing to inflammation in COPD. However, M2 polarization increases with disease severity, and those with severe COPD are observed to have much higher percentage of M2 polarization. Therefore, although M1/M2 phenotype is mostly non-polarized in healthy alveolar macrophages, there is markedly increased polarization in both M1 and M2 phenotypes, which can even be co-expressed in the same macrophage in COPD [185].

### 5.3. Inflammaging and Oxidative Stress in COPD

Several studies have found that COPD patients have accelerated telomere shortening in leukocytes compared to regular smokers, and morbidity of the disease has been correlated to shorter telomere length [186,187,188]. When looking specifically at relative telomere length in airway epithelial cells from COPD patients, one study found no correlation of shorter telomere length with the disease. However, cigarette smoke was shown to exacerbate telomere dysfunction with age, and this was linked with the onset of emphysema [189]. Interestingly, another research showed that the relative telomere length was increased with age in the lung tissue of COPD [190]. Bartling et al. confirmed that COPD negatively impacts the proliferation capacity of fibroblasts; however, they were unable to elucidate an association between this negative impact and telomere length [191]. While the majority of recent data indicates correlation with shortened telomere length and COPD, it is likely an inconsistent and unreliable biomarker as of now.

Moreover, ROS generation further contributes to inflammation by inducing the upregulation of pro-inflammatory cytokines such as IL-1 and TNF-α (Table 1) [56,76]. ROS have also been implicated in COPD muscle autophagy, which contributes to skeletal limb muscle atrophy [192]. In addition to TNF-α, IL-17A and IL-22 have been found to be elevated in the plasma of COPD patients [193]. IL-17A production can be enhanced by IL-21 stimulation via STAT3 phosphorylation in neutrophils [194]. Cigarette smoke can stimulate IL-17A release, which contribute to the apoptosis of type II alveolar cells (Figure 4) [195]. IL-17 secretion leads to neutrophil recruitment, and IL-22 is involved in tissue protection and regeneration [193]. The increase in IL-17A and IL-22 was shown to raise the levels of MMP, which are responsible for tissue destruction [193].

Inflammaging has been found to accelerate the progression of COPD [74]. Oxidative stress contributes to inflammaging by reduction of SIRT1 activity, which results in increased acetylation of p53, NF-κB, and FOXO3 (Table 1) [77]. Wild-type p53 protects against oxidative stress via its antioxidant transcription factor activity under normal condition [46]. However, under oxidative stress, p53 may exhibit pro-oxidant properties [45]. p53 can also contribute to inflammation by TLR regulation [196]. Mutant p53 produces mutant proteins and activates NF-κB transcriptional activity, both of which contribute to inflammation [196,197]. Indeed, polymorphisms of p53 have been linked to the pathogenesis of COPD via apoptotic signaling and emphysematous changes [79]. The activation of p53 can also result in diminished mitochondrial function due to its inhibition of PGC-1α and PGC-1β transcription. Damage to the mitochondria is involved in the pathogenesis of COPD [78]. The affected airways of COPD patients feature increased levels of mitochondrial ROS and exacerbated inflammation [80,81]. Mitochondrial dysfunction has also been hypothesized as a driving force of carcinogenesis in COPD patients (Figure 4 and Table 1) [80].

## 6. Inflammaging in Diabetes

### 6.1. Type I vs. Type II Diabetes

Diabetes is a disorder present in multiple subtypes and is marked by high levels of glucose. This chronic elevation in glucose leads to serious complications including tissue damage and organ failure [198]. The two key subdivisions of diabetes that will be highlighted in this article are Type I and Type II diabetes. Type I diabetes is characterized by complete shortage of insulin secretion due to the destruction of β cells in the pancreas by the immune system. Type II diabetes is much more common than type I, which is featured by insulin resistance [198]. Genetic component plays an important part in the development of Type II diabetes. Other risk factors for insulin resistance are obesity, lack of exercise, pregnancy, and excessive hormone secretion (Table 1) [85]. The diabetic life expectancy is six years shorter than that of the average healthy individual. Oxidative stress and endoplasmic reticulum stress have been shown to play critical roles in aging and Type II diabetes. These biological stresses are linked with cellular senescence, and contribute to the production of pro-inflammatory factors by senescent cells [199,200].

### 6.2. Role of Oxidative Stress in Diabetes

**Figure 5:**
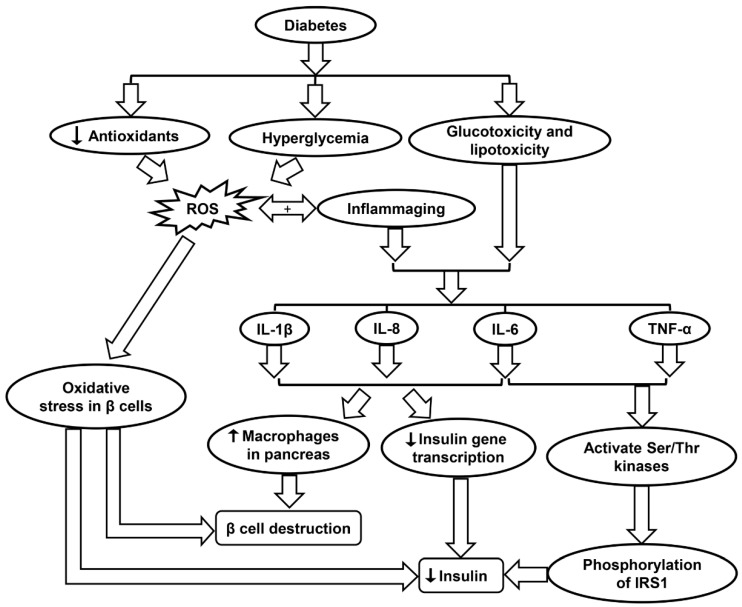
Schematic showing the molecular mechanisms contributing to diabetes progression. IL-1β, interleukin-1β; IL-8, interleukin-8, IL-6, interleukin-6; IRS1, insulin receptor substrate 1; ROS, reactive oxygen species; TNF-α, tumor necrosis factor-α.

Oxidative stress is directly influenced by glucose fluctuations, which are pivotal in the pathogenesis of diabetes [201]. According Monnier et al., glucose fluctuations after meals (postprandial periods), or more broadly, any type of glucose oscillation, elicited greater oxidative stress than chronic hyperglycemia (Figure 5 and Table 1). Length and severity of chronic hyperglycemia as well as the acute glucose shifts that occur regularly are the two main components when discussing glycemic disorders. In the study, the first component was integrated by hemoglobin (Hb) A1c. The second component was analyzed with newer devices that is able to detect glucose levels on an ambulatory basis. The results show that the mean urinary excretion rate of 8-iso-PGF2α (F2-isoprostane 8-iso-prostaglandin F2α), a key biomarker of oxidative stress, was significantly elevated (*p* < 0.001) in patients with Type II diabetes as compared to control [201]. Another study by Ceriello, et al. confirmed that acute glucose swings are more harmful to endothelial cells than sustained hyperglycemia, even when the subject in the latter group was exposed to a greater total amount of glucose over a 24 h period. This may have relevance to past studies that suggest greater activation in oscillating glucose pathways relating to protein kinase C, NADPH, inducible nitric oxide synthase (iNOS), and other inflammatory markers as opposed to sustained hyperglycemia [202].

When considering both type I and II diabetes, sustained hyperglycemia along with other metabolites such as free fatty acids has been implicated in the complications related to the nervous system, vascular endothelium, and kidneys [85]. Many of these complications may be the result of various stress-activated signaling pathways including NF-κB, p38 MAPK, and NH2-terminal Jun kinases, and other stress-activated protein kinases. It is widely accepted that elevated glucose levels result in oxidative stress due to the upregulation of mitochondrial ROS, glycation of proteins, and the autooxidation of glucose. Such processes may harm enzyme activity and cellular machinery (Table 1) [85]. Increased levels of free fatty acids also result in mitochondrial uncoupling and β-oxidation, ultimately causing more severe oxidative stress in the body. Advanced diabetes is also characterized by decreased levels of the antioxidants such as vitamin E and α-lipoic acid along with SOD, an enzyme that has important implications in the inactivation of the O_2_^•−^ radical (Figure 5 & Table 1). Other issues in diabetes including nephropathy, retinopathy, neuropathy, and vascular damage possibly correlate with a deficit in erythrocyte catalase, which removes H_2_O_2_ from tissues [85]. When looking specifically at diabetic kidney disease, multiple pathways in the kidney that produce ROS appear suspect, including glycolysis, polyol, as well as uncoupling of nitric oxide synthase, XO, and NOX. The body and its cells must be able to regulate glucose transport across membranes to maintain homeostasis, but this is often impossible in certain cells such as retinal capillary endothelial cells, renal mesangial cells, and neuronal and Schwann cells in the peripheral nervous system, along with other cells in the kidneys [203].

In diabetes, hyperglycemia induces the production of ROS. For Type II diabetes in which β cells are still intact and functional, the presence of ROS may cause oxidative stress in the β cells, leading to lower levels of insulin secretion (Figure 5 and Table 1). One type of ROS of particular interest is O_2_^•−^, which has been shown to be elevated both in vitro and in vivo studies of diabetes [86]. O_2_^•−^ is highly reactive and can be converted into H_2_O_2_ by mitochondrial SOD. O_2_^•−^ generation due to high glucose levels in diabetes also triggers multiple pathways such as enhanced polyol formation, increased hexosamine pathway flux, and activation of the protein kinase C isoform (Table 1) [86]. A study by Lortz and Tiedge studied antioxidant activity in diabetes and found that overexpressing SOD and catalase can shield pancreatic islets from ROS and maintain insulin production. Similarly, overexpression of GPX has been shown to protect INS-1 cells (an insulin secreting β cell-derived line) from ROS and reactive nitrogen species (RNS) attack (Table 1) [89].

### 6.3. Inflammaging and Diabetes

An increasing amount of evidence suggests that moderate inflammation precedes various age-related diseases, including type II diabetes mellitus. An important change associated with the onset of diabetes is vascular aging (Table 1) [199]. Vascular aging pertains to enlargement of vessels, thickening, stiffness, and compromised endothelial barrier strength, all of which have been recently suggested to be tied to pro-inflammatory factors. SASP genes like IL-1α, IL-1β, IL-6, and TNF-α are constantly activated in the diabetic body [199]. Endothelial cells and immune cells in diabetes are the targets of inflammation in conjunction with epigenetic modifications, but the bulk of inflammation related to vascular issues are the aforementioned SASP factors as well as the vascular cell adhesion molecule-1 (VCAM-1). Cellular senescence as well as the part it plays in vascular dysfunction and inflammation is a topic that is currently being extensively researched [199].

Inflammation may even influence the development of diabetes in an otherwise healthy individual. Hotamisligil et al. showed that insulin signal transduction can be disrupted by inflammatory cytokines, resulting in insulin resistance [87]. As an example, TNF-α and IL-6 activate multiple Ser/Thr kinases, which then catalyze serine phosphorylation of insulin receptor substrate 1 (IRS1). The phosphorylation of IRS1 disrupts its capability to mobilize phosphatidylinositol-3-kinase and Akt, causing disturbances in the body’s insulin processing mechanism (Table 1). Ser/Thr kinases such as JNK and IKK-b are two important factors that affect insulin signaling, further indicating that inflammatory tracks in bodily tissues can foster the inception of Type II diabetes [87]. Furthermore, inflammation may prompt the breakdown of β-cells. IL-1β, IL-6, and IL-8 production is increased in pancreatic islets due to glucotoxicity and lipotoxicity in diabetes. The presence of these pro-inflammatory molecules decreases insulin gene transcription and increases the presence of macrophages in the pancreas, ultimately inducing β-cell apoptosis (Figure 5 and Table 1) [87]. Anti-inflammatory treatments that may improve such conditions as diabetes include limited caloric intake, SIRT1 activators, and p38-MAPK inhibitors (Table 1) [88].

## 7. Inflammaging in Rheumatoid Arthritis (RA)

### 7.1. RA in Aging Population

RA is a chronic inflammatory disorder that affects joints by causing swelling, tenderness, and pain. It progresses to a targeted destruction of synovial joints which causes disability and early mortality. RA is also considered an autoimmune disease because of the contribution of inflammatory response to disease progression [204]. In this disease, the synovial membrane is infiltrated by overactive immune cells. Chronic inflammation can lead to tissue destruction with the deterioration of cartilage and bone. In a study by Helmick et al., it was reported that 21% of adults aged 18 years or older in United States (46.4 million) have arthritis, with 1.3 million of them having RA. Prevalence of the disease increases with age and affects the female population more than the male. Over 60% of the cases were reported by women, and even when adjusted for age, women accounted for 24% versus 18% for men [205]. Mortality among RA patients was also observed to be much higher than the general population. Gabriel et al. reported that RA patients had a standardized mortality ratio (SMR) of 1.27 with individual SMR scores for female and male being 1.41 and 1.08 respectively. This study focused on the population of Rochester, Minnesota with a cohort of patients who were picked from 1955–1994 and was monitored until the year 2000. New studies should be performed to evaluate the effects of modern medicine on mortality in RA patients [206].

### 7.2. Causes of RA

The cause of RA is very complex. It is believed to be affected by the person’s genotype, certain triggers from the environment such as cigarette smoke and infectious agents, and sometimes unknown factors (Table 1) [90]. Human leukocyte antigen (HLA)-DRB1 locus has been observed in those who test positive for having rheumatoid factor, an autoantibody which is found in most cases of RA. Sequence motifs such as the QKRAA sequence in the HLA-DRB1 region have been seen to increase the susceptibility of RA [90]. The autoimmune responses could be subsequently triggered by T cell selection, peptide affinity alterations, or changes in antigen presentation. In addition to the autoantibody rheumatoid factor, the anti-citrullinated protein antibody (ACPA) is often observed in RA patients as well [90]. There have been many risk alleles observed in those who are positive for ACPA, such as HLA-DRB1 and REL alleles. These can aid in the pathogenesis of RA either through T cell activation or through the NF-κB pathway, to promote inflammation and an autoimmune response (Table 1) [90,91]. Many gene-environment interactions can activate pathogenesis by affecting susceptible genes or by inducing epigenetic modifications. For example, those who have HLA-DR4 alleles have an increased chance to develop RA only if they smoke. Certain infectious agents such as the Epstein–Barr virus or cytomegalovirus have been linked to the development of RA, although the mechanisms are often not fully understood. This alteration of the immune system induces the production of antibodies that target host cells to cause synovial inflammation as well as cartilage and bone damage [90].

### 7.3. Inflammaging and the Innate Immune System in RA

**Figure 6:**
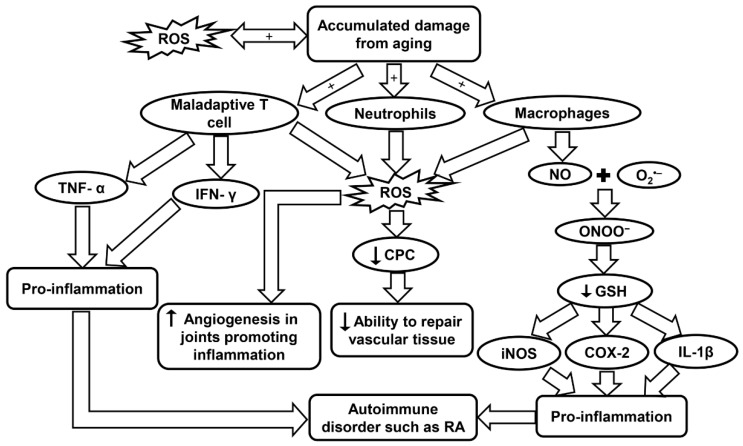
Schematic summarizing the molecular pathways contributing to RA. COX-2, cyclo-oxygenase 2; CPC, circulating progenitor cells; GSH, glutathione; IFN-γ, interferon-γ; iNOS, inducible nitric oxide synthase; IL-1β, interleukin-1β; RA, rheumatoid arthritis; ROS, reactive oxygen species; TNF-α, tumor necrosis factor-α.

Inflammation is a common symptom in age-related diseases and is considered to be a mechanism of innate immunity. The innate immune system is the body’s first line of defense in the detection of foreign bodies. It is believed that aging may be the result of accumulation of damage to DNA from sources such as oxidation. Damaged DNA may affect transcriptional signaling pathways and cause dyshomeostasis. Double-stranded breaks also tend to increase with aging and have been noticed to affect memory T cells more than naive T cells. In a RA patient, maladaptive T cells show signs of premature aging. Maladaptive T cells exhibit features such as the accumulation of CD28, attrition, telomere fragility, decreased ability to repair damaged DNA, and excessive cytokine production [92,207]. Throughout the life course, the continuous division, differentiation, and stimulus from foreign infectious microbes may cause the T cells to have a diminished diversity, which in turn limits their effectiveness. Regulatory T cells also go through transformations in the aging population. Naive-like regulatory T cells were reported to decrease while memory-like regulatory T cells actually increased with age. These regulatory T cells promote anti-inflammation by controlling the T cell compartment [92,208]. Maladaptive T cells obtained from RA patients have also been observed to not enter cellular senescence, and instead remain highly active, secreting pro-inflammatory cytokines such as TNF-α and IFN-γ. This aids in promoting inflammation and leads to an autoimmune disorder (Figure 6 and Table 1) [92,93,94]. Inflammation in RA is caused by a multitude of mechanisms shifting away from homeostasis which leads to uncontrolled chronic inflammation.

### 7.4. Inflammaging and Oxidative Stress in RA

Free radicals have proven to be an important aspect for consideration due to their association with inflammaging in RA. In a study by Mateen et al., RA patients were observed to have a higher rate of formation of ROS, as well as, lipid peroxidation, DNA damage, and protein oxidation. It was concluded that the oxidative stress may be linked with the chronic aspect of the disease [209]. In a population study by Kundu et al., synovial fluid was obtained from RA patients and the cellular components were isolated. Neutrophils, macrophages, and lymphocytes were identified as the major contributors in the pathogenesis of the disease. These cells generate ROS through NOX, a membrane-bound enzyme. Oxidative stress may cause abnormalities in signaling and proliferation of T lymphocytes that aid in the pathogenesis as explained earlier (Table 1). It was also observed that neutrophils were producing three times more ROS than macrophages. Macrophages, however, produced high levels of NO, which has an important role during the oxidative burst seen in inflammation [95].

NO vital function in chronic inflammation and autoimmunity was measured in RA patients. A significant increase in NO levels was observed in RA patients which suggests that the synovium and other inflamed tissues are responsible for this endogenous source of NO [210]. NO can combine with O_2_^•−^ produced by NOX to create ONOO^−^. This toxic molecule causes rapid protonation which in turn causes severe decrease of many anti-oxidants with –SH groups such as GSH. When GSH decreases in the body it activates many inflammatory mediators such as cyclo-oxygenase 2, cytosolic phospholipase A2, IL-1β, iNOS, and TNF-α through the NF-κB-signaling pathway (Figure 6 and Table 1). The oxidant status in the inflamed areas of a patient may be evaluated effectively by measuring the O_2_^•−^ levels in the peripheral blood [96].

In a study by Jaswal et al., it has also been seen that blood GSH and total thiols were significantly lowered in RA patients, and conversely, malondialdehyde, a marker of oxidative stress, was at a much higher level. The same study also reported that plasma vitamin C, which is an antioxidant parameter, was also present in much lower concentrations in RA patients and could be used as an early indicator of oxidative stress. An antioxidant therapy has been found to help RA patients recover, but more clinical trials are needed to evaluate this treatment [211]. High ROS levels and low antioxidant defenses have been observed to have a major role in decreased levels of circulating progenitor cells (CPCs) in RA patients. The lower level of CPCs affects the body’s ability to repair vascular tissue (Figure 6) [212]. CD34^+^ cells, which are precursors for endothelial, smooth muscle, and myocardial cells in the cardiovascular system, are recruited to the inflamed synovia, further depleting the CPC in the circulatory system and increasing the probabilities of developing CVD [212]. Although the body’s ability to repair vascular tissue is diminished, the formation of new blood vessels near the hypertrophic joints was actually seen to increase in RA. Angiogenesis is caused by the increase of pro-angiogenic factors that are secreted from the cells present in the synovial tissue. Angiogenesis plays a critical role in many chronic inflammatory diseases such as RA because it provides O_2_ and nutrients to the hypertrophic joint as well as promoting inflammation and bone destruction [213]. Leptin, a hormone secreted by adipose tissue, has been observed to play an important role in both autoimmune and inflammatory rheumatic diseases. Leptin can induce ROS formation and aid in inflammation by causing angiogenesis, while inhibition of this hormone could be a therapeutic treatment in RA to decrease angiogenesis and thereby infiltration as well (Table 1) [97].

## 8. Potential Treatment for Inflammaging-Related Diseases

### 8.1. Drugs

**Figure 7:**
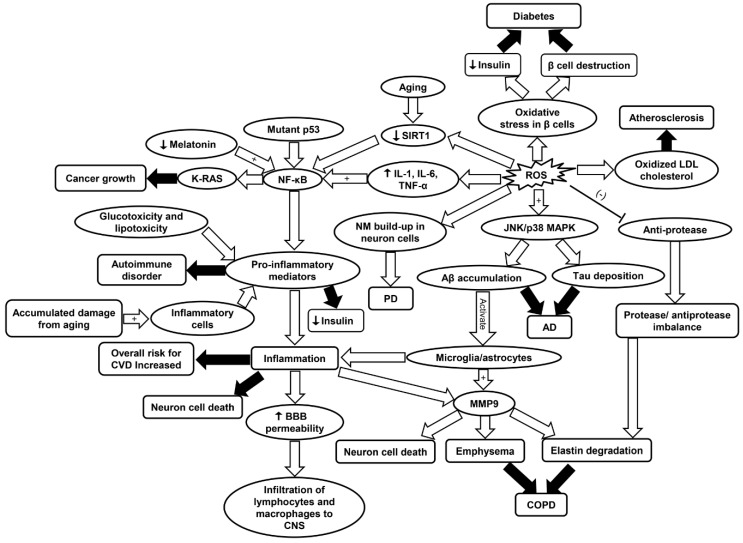
Schematic summarizing the primary molecular signaling involved in different diseases including CVD, cancer, AD, PD, COPD, diabetes, and RA. AD, Alzheimer’s disease; Aβ, amyloid beta plaques; BBB, blood brain barrier; COPD, chronic obstructive pulmonary disease; CVD, cardiovascular disease; IL-1, interleukin-1; IL-6, interleukin-6; JNK/p38 MAPK, c-Jun N-terminal kinase/p38 mitogen-activated protein kinase; LDL, low density lipoprotein; MMP9, matrix metalloprotease 9; NF-κB, nuclear factor kappa-light-chain-enhancer of activated B; SIRT1, sirtuin-1; PD, Parkinson’s disease; ROS, reactive oxygen species; TNF-α, tumor necrosis factor-α.

As discussed, inflammaging and oxidative stress are associated with age-related diseases such as CVD, COPD, neurodegenerative diseases, cancer, diabetes, and RA. A complete review of the molecular mechanisms involved in different diseases is shown in Figure 7. Immunosenescence, detailed in this review, has been described as a culprit of inflammaging and there are reports of therapeutic success with vaccination [214]. Adjutant supplemented antigen promotes a robust immune response in subjects above 80 years old, thus attenuating immunosenescence [215]. Moreover, according to a comprehensive review by Xia et al., epimedium total flavonoids (EF) and icariin (Ica) are among many intervention models for inflammaging. Icariin, a natural flavanol glycoside, enhanced SIRT6 enzyme expression and repressed NF-κB inflammatory signaling pathways [216].

Moreover, metformin inhibits the mammalian target of rapamycin (mTOR) pathway, activates AMPK, and reduces ROS levels, insulin and insulin like growth factor-1 signaling [50]. Zinc supplements and vitamin E are confirmed potent modulators of inflammaging [217]. In addition to zinc, vitamin E, vitamins C, D, carotenoids, and polyphenols, have been demonstrated to have antioxidant properties. They counteract the activation of proinflammatory mediators, NF-κB, MAPK, and activator protein-1. These macronutrients are particularly effective in lowering the risk of COPD (Table 1) [16,83].

Deteriorating heart function and neuronal loss as a result of aging can be potentially treated by resveratrol, a SIRT1 activator [218]. However, more potent SIRT1 activators have been identified (e.g., SRT1720). In obese mice, these activators enhance insulin response and upregulate mitochondrial function and population [218]. A study assessing SIRT1 as an intervention for inflammaging suggests that SIRT1 activators and polyphenols offer a potential therapy for COPD (Table 1) [82]. SIRT1 is downregulated in the lungs of COPD patients and this reduction is associated with increased proinflammatory species [219]. It has been shown that melatonin decreases the progression of senescence [220]. Melatonin, synthesized from 1-tryptophan and present in some plants, also expressed in the human immune system, has antioxidant properties. Therefore, metformin and melatonin have been established not only as drugs that counteract age-related diseases, but also as drugs with corrective measures against the pathophysiology of aging [50].

The phosphorylation of the protein NF-κB is inhibited by 3-bromo-4, 5-dihydroxybenzaldehyde (BDB), a natural substance produced by red algae Polysiphonia morrowii. BDB also impedes the production of IL-6 making it an effective anti-inflammatory substance [37]. In mice, cardiac function recovery after myocardial infarction was enhanced after administration of BDB. Mortality and infarcted size were also attenuated by the administration of BDB (Table 1) [37]. Since inflammatory response can be modulated by BDB, it is a possible target for therapeutic intervention in patients who have suffered from myocardial infarction. Active fraction combination from Liuwei Dihuang decoction (LW-AFC) is derived from Liuwei Dihuang (LW), a traditional Chinese herbal medicine [65,221]. Among its therapeutic effects, it increased longevity, reduced senescence, reverted the decreased levels of helper and suppressor T cells and B cells. It also curbed aberrant secretions of interleukins and downregulated inflammation in senescence accelerated mouse resistant 1 strain [222]. It is, therefore, a potential anti-inflammaging drug.

IFNβ1a is an anti-inflammatory cytokine that has shown effectiveness in attenuating inflammation and reversing cognitive impairment in AD rats [64]. Additionally, considering the critical roles of NF-κB in stimulating neuroinflammation, the inhibitors for NF-κB such as phytochemicals and polyphenol-containing compounds, represent a promising AD therapy [12]. Substances with antioxidant properties including lipoic acid and GSH were found to be able to protect neuron cells in a rat model of PD (Table 1) [62]. However, other studies have indicated that administration of antioxidants did not show effectiveness in protecting against neurodegeneration. This is probably because that the exogenous antioxidants are lack of specificity and may interrupt cellular redox signaling [62]. Also, it has been established that melatonin decreases the progression of senescence and may delay neurodegenerative progression [220]. Melatonin was found to prevent striatal degeneration and improve motor function in a rat model of PD as well as reduce the Aβ-induced neurotoxicity in rats (Table 1) [72,73]. Canakinumab is a human anti-monoclonal antibody which has been shown promising in treating RA in clinical trials [99]. It led to the decline of reoccurrence of cardiovascular episodes (Table 1) [223,224]. Further, it targets the IL-1β innate immunity pathway, thus playing an anti-inflammatory role [223].

### 8.2. Stem Cell Interventions

To counteract immunosenescence, rejuvenation of the T-cell mediated immune system has been proposed through replacement, reprograming, and restoration. These are achieved by autologous blood transfusion, pharmacologically enhancing telomerase activity, autologous hematopoietic stem cell (HSCs) transplantation, and pharmacological normalization of thymic function, respectively [225]. Inflammatory signaling in hematopoietic aging is determined by the nature of changes in immune system as aging progresses [226]. Also, aging of the hematopoietic system results in immunosenescence. As a result, manipulation of HSCs has been proposed as a countermeasure to aging associated diseases. Such methods include HSC reprogramming into induced pluripotent stem cells subsequently subjecting these cells to re-differentiation into juvenile HSC [227]. Another technique involves targeting mTOR, a nutrient sensing protein, implicated in the remodeling of the hematopoietic system [228,229]. Considering the phenomenon of stem cell exhaustion as a consequence of aging, the use of mesenchymal stem cells from juvenile animals has been proposed as a therapeutic remedy for age-related diseases. This is especially the case for COPD which has been shown to be associated with cellular senescence [230].

### 8.3. Diet

While drugs and stem cell interventions may open a window to therapeutic measures for inflammaging, diet has been proposed to play a consequential role in the epigenetics of inflammaging. Metabolites, phytochemicals, micronutrient also contribute to epigenetic modulation and diminish the risks of age-related diseases and inflammaging. However, the response of epigenetic programing to comparable diet varied between individuals [231]. Although yet to be verified in humans, caloric restriction has been shown to be beneficial to the onset of immunosenescence in animal models [217]. Furthermore, various linked paths associated with carcinogenesis, inflammatory response, such as the activation of NF-κB and eicosanoids pathway can be modulated by Mediterranean diet [47]. Endothelial progenitor cells (EPCs) play a critical role in neo-angiogenesis and vascular aging. It was found that Mediterranean diet nutraceuticals regulate the population and the physiological condition of EPCs, thus mitigating the process of inflammaging in the cardiovascular system (Table 1) [38]. Also, the consumption of diets such as whole grains cereals, vegetables, fish, and fruits have been shown to have protective, antioxidant, and anti-inflammatory effects [16,83]. Immunosenescence involves alterations in T cell immunity typified by population decline of naive T cell and a heightened CD28- memory T cell subset population and differentiation [232]. Dietary intervention and fasting both affect autoimmunity and immunosenescence, through T cell regulation. Thus, targeting T cell function by dietary means delivers alternative therapeutic remedy for autoimmune pathologies [233].

### 8.4. Plant Supplements

Additionally, the reduced form of histone deacetylase 2 (HDAC2), which plays a role in transcriptional regulation and cell cycle progression, can promote DNA damage, cellular senescence, and steroid resistance. Decrease levels of HDAC2 have been observed in the lungs of COPD patients [84]. This reduction occurs through ubiquitination-proteasome dependent degradation, especially due to oxidative stress resulting from smoking cigarettes. The reduction of HDAC2 is not only associated with inflammation, but also, steroid resistance. Nevertheless, dietary and plant derivatives can target HDAC2, and abolish steroid resistance in COPD patients. Theophylline, baicalin, quercetin, and erythromycin have all been confirmed to target HDAC2 [84]. Baicalin, for instance, is an anti-inflammatory flavonoid derived from Scutellaria baicalensis, a plant indigenous to some countries in the Far East. It interacts with HDAC2 and inhibits phosphorylation, thus, alleviating steroid resistance (Table 1) [84].

Sulforaphane, present in cruciferous vegetables such as broccoli sprouts, possesses anticarcinogenic and antioxidant functions [58,59]. Medicinal anti-inflammatory plants have shown promising results in inhibition of inflammaging, and thus exert positive effect on cardiovascular health (Table 1) [35]. With ample species and their corresponding functions detailed by Shayganni et al., for example, the roots of Scutellaria baicalensis possess anti-inflammatory properties and reduce inflammatory factors such as IL12, TNF-α, and NF-κβ [35,234]. In a comparative study, a higher anti-inflammatory and antidyslipidemic action by fenofibrate was observed in RA patients in comparison to statins (Table 1) [98]. Furthermore, present in fruits such as berries and vegetables, the defensive effect of polyphenols on age related-diseases have been well documented. They have been shown to play a role in the prevention of age-related diseases such as cancer and cardiovascular diseases (Table 1) [36].

### 8.5. Gut Microbiome

An interesting area of research in inflammaging is the role of the gut microbiome. Implicated in inflammation and cancer, human intestinal microbiota influences inflammatory elements such as TNF, IL-6 and IL-8, and, the levels of these inflammatory elements increase with aging. This increase is associated with immunosenescene and inflammaging [10]. Relatively new remedies, such as fecal microbiota transplantation have proven to be effective in the therapy and prevention of age-related diseases such as type II diabetes and atherosclerosis (Table 1) [39]. In fact, poor microbiota has been associated with immunosenescence and inflammaging. Dysbiosis of the microbiota may lead to decreased immune function, consequently immunosenescence and inflammaging. The restoration of microbiota equilibrium and homeostasis is therefore a therapeutic target for immunosenescence and inflammaging. Type II diabetes and colorectal cancer are diseases that can be treated by such therapy [11]. In fact, hyperglycemia and dyslipidaemia, besides leading to “garb-aging”, also promote epigenetics and microbiota remodeling [87].

Finally, studies have shown that LW-AFC, a new formula derived from LW, influences the gut microbiome of senescence-accelerated mouse prone 8 strain, a mouse model of AD [221]. It was further reported that administration of LW-AFC can attenuate the cognitive impairment and improve the neuroendocrine-immune system in AD mice (Table 1) [65]. This further suggests the role of intestinal microbiome in aging, inflammation, and immunosenescence. Notwithstanding, while all these findings are significant milestones in combating inflammaging, more research is needed for more effective and readily available medical intervention.

## 9. Summary and Future Perspective

While inflammaging have recently received modest attention, more research is needed to fully elucidate its underlying mechanisms for the advancement of therapeutic intervention for the diseases associated with it. Here we elaborate the implicated role of inflammaging in multiple disease states that are more prevalent among aging individuals. As we have shown, inflammaging is associated with the decline of vascular function, which is a critical risk factor for the onset of CVD. Furthermore, oxidative stress as a result of the decline of cellular proliferation, senescence, and loss of adaptive immune function, immnunosenescence, is characteristic of age-onset diseases such as CVD. Increased mitochondrial ROS and enhanced production of pro-inflammatory cytokines due to aging are implicated in neurodegenerative diseases and cancer tumorigenesis. Other prominent diseases among the elderly such as RA and COPD can be exacerbated by aberrant intracellular signaling, maladaptive immune response, and damaged DNA due to oxidative stress. However, type II diabetes preceded by moderate inflammation, can exacerbate oxidative stress influenced by the oscillation of glucose levels, thus, accelerating inflammaging. Taken together, inflammaging plays a significant role in the pathogenesis of aforementioned diseases, some of which are the leading causes of death among the elderly. ROS appear to have a direct linkage with inflammaging and cell senescence. Age-related oxidative stress and attenuated antioxidant defense possibly contribute to the progression of most diseases discussed in this paper. However, the following questions remain unresolved in this area that may need further exploration. First, does age-induced antioxidant (e.g., GPX3) decrease create a prothrombotic environment contributing to CVD occurrence? Second, what are the roles of mtDNA mutation and mitochondrial dysfunction in cancer development? Third, why most of the current antioxidant therapies fail to fully reverse the condition of oxidative disorders? Fourth, what is the exact relationship between shortened telomere length and age-related diseases? Aging is accompanied with complex biological alterations in respect of redox condition and immune environment, which may render the aging population more susceptible to environmental risk factors. An in-depth understanding of its underlying mechanisms will be of tremendous benefit to the development of more effective medical intervention for the pathophysiology of several geriatric diseases.

## Figures and Tables

**Table 1:** Causative factors, roles of inflammation, oxidative stress, and treatment strategies of age-related diseases including CVD, cancer, neurodegenerative diseases, COPD, diabetes, and RA.

Disease	Causative Factors	Roles of Inflammaging and ROS		Potential Treatments
CVD	Vascular dysfunction [23] Aging [21] Immunosenescence [24]	ROS oxidize LDL cholesterol → oxidized LDL is phagocytosed by macrophages → macrophages release pro-inflammatory mediators and ROS → cause further LDL oxidation → atherosclerosis [25,26] MPO → promotes oxidation of LDL and limit NO availability → vascular dysfunction [27] Decreased NO → leads to decline in SOD3 levels → cause age-related vascular dysfunction [28] O_2_^•−^ reacts with NO → form ONOO^−^ → endothelial cell damage [26] XO induced O_2_^•−^ formation → interacts with EGFR → induces vascular remodeling → CVD [26] Senescent T cells → release effector molecules → stimulate release of cytokines such as IFN-γ → amplify inflammation [29] IL-1β and IFN-γ → induces M1 phenotype → increases arterial plaques [30] CCL-2 → activates ERK1/NF-κB pathways → increases atherosclerotic plaque formation [30] SIRT1 coupled with Rel/p65 → inhibits NF-κB pathway → reduces systemic inflammation and impaired vessel dilation function [31] Decrease in telomerase activity in plaques → telomeric exhaustion → accumulation of senescent endothelial cells → VSMC proliferation → vascular remodeling [24] LTL shortening → increased senescent epithelial cells → pathogenic vascular remodeling [32]		SIRT1 activator [33,34] Medicinal anti-inflammatory plant [35] Polyphenols [36] BDB [37] Mediterranean diet [38] Fecal microbiota transplantation [39]
Cancer	Oxidative stress [40]	ROS → activate p38 MAPK → initiates/blocks tumor development based on degree of activation and cancer type [41,42] ROS → activate NRF2 → anabolic purine synthesis pathways → tumor cell proliferation [43] Oxidative stress → activates p53 pathway → induces tumor cell apoptosis [44,45,46] Inflammaging → increased levels of pro-inflammatory cytokines including IL-1, IL-6, and TNF-α → stimulate ROS formation → DNA mutagenesis → tumorigenesis [46,47,48,49,50] Mitochondrial ROS → upregulate IL-1, IL-6, and TNF-α → activates NF-κB signaling → increased oncogenic K-Ras levels → cancer progression [51,52,53,54,55,56] G-MDSCs → produce ROS → suppress CD8^+^ T cells → promote tumor growth and progression [57]		Sulforaphane, present in cruciferous vegetables such as broccoli sprouts [58,59] Mediterranean diet [47] Polyphenols [36]
Neurodegenerative Diseases	Aging	Age, AD, or trauma → increased ROS formation → causes KCNB1 oxidation → impairs hippocampal function and leads to neurodegeneration [60] Inflammaging → increased CCL11 levels in neurons → interrupt differentiation of Schwann cells → reduced regenerative capacity of aged nerves [61]		
	AD	Intracellular deposition of NFT and extracellular accumulation of Aβ plaques [62]. Oxidative stress [16]	Aβ deposition in astrocytes and microglia → triggers inflammatory response → excessive ROS formation → activates JNK/p38 MAPK pathways → leads to Aβ accumulation and Tau hyper-phosphorylation [12,16,63] Aβ accumulation → depletes Ca^2+^ in ER, damage mitochondrial and plasma membrane → further ROS formation → induces neuron cell death [62] Increased ROS or decreased SIRT1/melatonin → activates NF-κB pathway → induces the expression of pro-inflammatory genes [12]	Phytochemicals and polyphenol-containing compounds [12] IFNβ1a [64] LW-AFC [65]
	PD	Loss of dopaminergic neurons in the SNpc area of the brain [66,67] Intracellular deposition of misfolded α-synuclein [66,67]	Aging, environmental toxicities, oxidative stress → buildup of NM → neuron cell death Complex I interruption and mitochondrial dysfunction → increased ROS accumulation → leads to oxidative damage on lipids, proteins, and DNA → neuron cell death [62,68] Pro-inflammatory cytokines such as IFN-γ, TNF-α or TLR activation → activates microglia → induce the release of ROS, nitrogen species, MMP, and pro-inflammatory cytokines [69,70] Inflammaging → increases inflammatory cytokines → favor BBB permeabilization → infiltration of lymphocytes and macrophages to CNS [69,70,71]	Lipoic acid and GSH [58] Melatonin [72,73]
COPD	Cigarette smoke [74] Toxic gases [74]	Oxidative stress → inactivates anti-proteases → causes protease/anti-protease imbalance → emphysema and elastin degradation [75] ROS → increase pro-inflammatory mediators such as IL-1 and TNF-α → inflammation [56,76] Oxidative stress → reduces SIRT1 activity → acetylation of p53, NF-κB, and FOXO → results in inflammation [74,77] Activation of p53 → inhibits PGC-1α and PGC-1β transcription → diminished mitochondrial function → contributes to COPD pathogenesis and may contribute to carcinogenesis in COPD [78,79,80,81]		SIRT1 activators and polyphenols [82] Zinc, vitamin E, vitamins C, D, and carotenoids [16,83] Pharmacological and plant elements such as theophylline, sulforaphane, nortriptyline, baicalin, quercetin, erythromycin, and curcumin [84]
Diabetes	Vascular aging Genetic insulin resistance [85] Obesity, lack of physical exercise, pregnancy, hormone excess [85] Oxidative stress [85]	Increased glucose levels, especially glucose fluctuation → leads to increased mitochondrial ROS formation and glycation of proteins → oxidative stress → decreased enzyme activity [85] Advanced diabetes → decreased antioxidants such as vitamin E, α-lipoic acid, and SOD → oxidative stress [85] ROS → cause oxidative stress in β cell → decreased insulin secretion [86] Elevated O_2_^•−^ formation → results in increased polyol activity, increased hexosamine pathway flux, and activation of PKC isoform → lead to β cell dysfunction [86] Inflammaging → increased TNF-α and IL-6 → activate multiple Ser/Thr kinase → catalyzes serine phosphorylation of IRS1 → disrupts the capability of IRS1 to mobilize phosphatidylinositol-3-kinase and Akt → disturbs insulin processing mechanism [87] Increased IL-1β, IL-6, and IL-8 in pancreatic islets → result in down-regulation of insulin gene expression and increase of macrophages in pancreas → leads to β-cell apoptosis [87]		SIRT1 activators [88] p38-MAPK inhibitors [88] Limited caloric intake [88] Antioxidants like SOD, catalase, and GPX [89] Fecal microbiota transplantation [39]
RA	Genotype Certain triggers from the environment such as cigarette smoke and infectious agents [90]	REL allele → leukocyte activation through NF-κB pathway → increase autoimmune response/inflammation [90,91] Maladaptive T cells → secrete pro-inflammatory cytokines such as TNF-α and IFN-γ → promote inflammation and leads to autoimmune disorder [92,93,94] Neutrophils, macrophages, and lymphocytes in RA → increased ROS formation → causes abnormalities in T cell signaling and proliferation [95] O_2_^•−^ reacts with NO → form ONOO^−^ → decreased GSH levels → activate NF-κB signaling pathways → increased inflammatory mediators such as cyclo-oxygenase 2, cytosolic phospholipase A2, IL-1β, iNOS, and TNF-α → promote inflammation and leads to autoimmune disorder [96] Adipose tissue → secrets leptin → causes angiogenesis → induces ROS expression and aids in inflammation [97]		Fenofibrate [98] Canakinumab [99]

AD, Alzheimer’s disease; Akt, protein kinase B; Aβ, amyloid beta plaques; BBB, blood brain barrier; BDB, 3-bromo-4, 5-dihydroxybenzaldehyde; CCL, CC chemokine ligand; CNS, central nervous system; COPD, chronic obstructive pulmonary disease; CPCs, circulating progenitor cells; CVD, cardiovascular disease; EGFR, epidermal growth factor receptor; ER, endoplasmic reticulum; ERK, extracellular regulated protein kinases; G-MDSCs, granylocytic-myeloid derived suppressor cells; GSH, glutathione; IFN, interferon; IP-10, IFN-γ-induced protein 10; IL, interleukin; iNOS, inducible nitric oxide synthase; IRS1, insulin receptor substrate 1; KCNB1, Voltage-gated potassium (K^+^) channel sub-family B member 1; LTL, leukocyte telomere length; LW-AFC, Active fraction combination from Liuwei Dihuang decoction; MMP, matrix metalloprotease; MPO, myeloperoxidase; LDL, low-density lipoprotein; MS, multiple sclerosis; NF-κB, Nuclear factor kappa-light-chain-enhancer of activated B; NFT, neurofibrillary tangles; NLRP3, pryin domain containing-3 protein; NM, neuromelanin; NRF-2, nuclear factor erythroid 2-related factor 2; PD, Parkinson’s disease; PGC, peroxisome proliferator-activated receptor-γ coactivator; PKC, protein kinase C; RA, rheumatoid arthritis; ROS, reactive oxygen species; SIRT1, Sirtuin-1; snPC, substantia nigra pars compacta; SOD, superoxide dismutase; Th1, T helper cell 1; TLR, toll-like receptor; TNF-α, tumor necrosis factor-α; VSMC, vascular smooth-muscle cells; XO, xanthine oxidase.

## Abbreviations

ACPA	Anti-citrullinated protein antibody
AD	Alzheimer’s disease
BDB	3-bromo-4, 5-dihydroxybenzaldehyde
CCL11	CC Chemokine ligand 11
COPD	Chronic obstructive pulmonary disease
CCL-2	Chemokine (C-C motif) ligand 2
CPCs	Circulating progenitor cells
CRP	C-reactive protein
CSF	Cerebrospinal fluid
CVD	Cardiovascular disease
EF	Epimedium total flavonoids
EGFR	Epidermal growth factor receptor
EPCs	Endothelial progenitor cells
ER	Endoplasmic reticulum
G-MDSCs	Granylocytic-myeloid derived suppressor cells
GPX	Glutathione peroxidase
GSH	Glutathione
Hb	Hemoglobin
HDAC2	Histone deacetylase 2
HSCs	Hematopoietic stem cell
Ica	Icariin
IL-1	Interleukin-1
iNOS	Inducible nitric oxide synthase
IP-10	Interferon–gamma induced protein 10
JNK	c-Jun N-terminal kinase
KCNB1	Voltage-gated potassium (K+) channel sub-family B member 1
KEAP1	Kelch-like ECH-associated protein 1
LDL	Low density lipoprotein
LO	Lipoxygenase
LRRK2	Leucine-rich repeat kinase 2 (LRRK2)
LTL	Leukocyte telomere length
LW	Liuwei Dihuang
LW-AFC	Active fraction combination from Liuwei Dihuang decoction
MAPK	Mitogen-activated protein kinase
MMP	Matrix metalloprotease
MPO	Myeloperoxidase
MS	Multiple sclerosis
mTOR	Mammalian target of rapamycin
mtDNA	Mitochondrial DNA
mtROS	Mitochondrial ROS
NF-κB	Nuclear factor kappa-light-chain-enhancer of activated B
NLRP3	Pryin domain containing-3 protein
NM	Neuromelanin
NO	Nitric oxide
NOX	NADPH oxidase
NRF2	Nuclear factor erythroid 2-related factor 2
PD	Parkinson’s disease
PFK	Phosphofructokinase
PINK1	PTEN-induced putative kinase 1 (PINK1)
PI3K	Phosphatidyl inositol 3-kinase
PGC-1α	Peroxisome proliferator-activated receptor-γ coactivator 1alpha
PPP	Pentose phosphate pathway
RA	Rheumatoid arthritis
ROS	Reactive oxygen species
SASP	Senescence-associated secretory phenotype
SMR	Standardized mortality ratio
SNpc	Substantia nigra pars compacta
STAT3	Signal transducers and activators of transcription 3
TGF-β	Transforming growth factor-β
Th1	T helper cell 1
TIGAR	TP53-induced glycolysis and apoptosis regulator
TLR	Toll-like receptor
TNF-α	Tumor necrosis factor alpha
VCAM-1	Vascular cell adhesion molecule-1
VSMCs	Vascular smooth-muscle cells
XO	Xanthine oxidase
8-iso-PGF2α	8-iso-prostaglandin F2α
8-oxodG	8-oxo-7,8-dihydro-2’-deoxyguanine
